# Feasibility of Follow-Up Studies and Reclassification in Spinocerebellar Ataxia Gene Variants of Unknown Significance

**DOI:** 10.3389/fgene.2022.782685

**Published:** 2022-03-25

**Authors:** Fatemeh Ghorbani, Mohamed Z. Alimohamed, Juliana F. Vilacha, Krista K. Van Dijk, Jelkje De Boer-Bergsma, Michiel R. Fokkens, Henny Lemmink, Rolf H. Sijmons, Birgit Sikkema-Raddatz, Matthew R. Groves, Corien C. Verschuuren-Bemelmans, Dineke S. Verbeek, Cleo C. Van Diemen, Helga Westers

**Affiliations:** ^1^ Department of Genetics, University Medical Center Groningen, University of Groningen, Groningen, Netherlands; ^2^ Department of Hematology and Blood Transfusion, Muhimbili University of Health and Allied Sciences, Dar es Salaam, Tanzania; ^3^ Shree Hindu Mandal Hospital, Dar es Salaam, Tanzania; ^4^ Groningen Biomolecular Sciences and Biotechnology Institute, Zernike Institute for Advanced Materials, University of Groningen, Groningen, Netherlands; ^5^ Structural Biology in Drug Design, Department of Drug Design, Groningen Research Institute of Pharmacy, University of Groningen, Groningen, Netherlands

**Keywords:** genetic diagnostics, variant of unknown significance, spinocerebellar ataxia, gene panel, functional studies, protein modeling, segregation

## Abstract

Spinocerebellar ataxia (SCA) is a heterogeneous group of neurodegenerative disorders with autosomal dominant inheritance. Genetic testing for SCA leads to diagnosis, prognosis and risk assessment for patients and their family members. While advances in sequencing and computing technologies have provided researchers with a rapid expansion in the genetic test content that can be used to unravel the genetic causes that underlie diseases, the large number of variants with unknown significance (VUSes) detected represent challenges. To minimize the proportion of VUSes, follow-up studies are needed to aid in their reclassification as either (likely) pathogenic or (likely) benign variants. In this study, we addressed the challenge of prioritizing VUSes for follow-up using (a combination of) variant segregation studies, 3D protein modeling, *in vitro* splicing assays and functional assays. Of the 39 VUSes prioritized for further analysis, 13 were eligible for follow up. We were able to reclassify 4 of these VUSes to LP, increasing the molecular diagnostic yield by 1.1%. Reclassification of VUSes remains difficult due to limited possibilities for performing variant segregation studies in the classification process and the limited availability of routine functional tests.

## Introduction

The autosomal dominant inherited cerebellar ataxias, also known as the spinocerebellar ataxias (SCAs), are a clinically and genetically heterogeneous group of rare disorders characterized by a late age of onset, progressive motor incoordination, gait disturbances, dysarthria, loss of balance and additional variable neurological symptoms (Manto, 2005; Shakkottai and Fogel, 2013). To date, 47 SCA types have been characterized, for which 38 causal genes have been identified (Coarelli et al., 2018; Huang and Verbeek, 2019). While the majority of SCA patients carry trinucleotide (CAG)n polyglutamine repeat expansions in the coding regions of a number of those genes, non-coding repeat expansions and conventional variants (single nucleotide variants and small indels) have also been identified in SCA genes (Hersheson et al., 2012). Studies in ataxia patients have already shown that using Next Generation Sequencing (NGS)-based approaches for conventional variant detection can lead to a notable increase in the diagnostic rate (Németh et al., 2013; Galatolo et al., 2018; Maturo et al., 2020). Although the advent of NGS has enabled parallel sequencing of known SCA genes and the detection of conventional variants, it has brought with it the challenge of interpreting large numbers of variants with unknown significance (VUSes). VUSes cause challenges for clinicians, patients and at-risk family members. Clinicians are often struggling whether VUSes should be disclosed to patients, as including VUSes in decision making is difficult with respect to clinical follow ups of patients and at-risk family members (Hoffman-Andrews, 2017). According to the American College of Medical Genetics (ACMG) guidelines, a VUS cannot be used in clinical decision making unless its clinical significance is resolved by follow-up studies (Ellard et al., 2020). A common follow-up study is to test other affected family members for segregation of the variant. However, this requires the availability, consent and cooperation of affected family members, which is not feasible in most cases. Functional studies can also be used to assess the effects of the candidate variants, but these studies are often time-consuming, considered expensive and labor-intensive and are therefore not part of standard genetic diagnostics (Di Resta et al., 2018). In addition, functional assays require a tailored readout for the functionally diverse SCA proteins, but for some of these proteins we do not know their exact function yet. Efforts to follow-up VUSes with alternative tests not traditionally performed by genetic labs such as protein modeling and functional tests are thus needed in a diagnostic setting in order to reclassify VUSes and reach (or exclude) definitive genetic diagnoses.

In this study, we addressed the challenge of prioritizing VUSes for follow-up in the context of SCA genetic diagnostics. We retrospectively screened a Dutch cohort of 368 cerebellar ataxia patients without a genetic diagnosis using a custom-made targeted gene panel that tests for the presence of single nucleotide variants and small indels in the 33 SCA genes and 3 novel candidate SCA genes known up to October 2017. Following diagnostic variant classification based on ACMG guidelines (Ellard et al., 2020), we present a procedure for prioritization of VUSes for follow-up studies that include segregation analyses, protein modeling and functional testing.

## Materials and Methods

### Patient Selection

We selected 368 seemingly unrelated patients with sporadic or familial cerebellar ataxia who were referred to the laboratory of the Department of Genetics, University Medical Center Groningen, Groningen, Netherlands for SCA genetic diagnostics. All these patients remained without a molecular genetic diagnosis after routine testing for the presence of a coding CAG repeat expansion in the SCA1, 2, 3, 6, 7 and 17 genes. The gene panel analysis presented in this study is in line with the original request for diagnostic testing, and therefore no additional informed consent was requested.

### Targeted Sequencing

A SureSelect targeted gene-capturing panel (Agilent Technologies, Santa Clara, CA, United States) was designed for the enrichment of 36 SCA genes (Supplementary Table S1). At the time this study was initiated (October 2017), 33 conventional SCA genes were known (Coutelier et al., 2017). In addition, we included three putative novel genes without a designated SCA symbol (*FAT1*, *EP300* and *KIF26B*) that had been previously reported by our group (Nibbeling et al., 2017). The panel was designed to screen all the coding exons (including the intron-exon boundaries), promoters and 3′UTRs of these genes. Currently, variants in the promoters and 3′UTRs are not part of standard SCA molecular diagnostics in our hospital. Since the goal of this work is to re-evaluate VUSes detected in standard current diagnostics, the data on variants in promoter regions and 3′UTRs are outside of the scope of the current study.

The sequences were uploaded into the probe design tool eArray (Agilent Technologies) based on the human assembly GRCh37 (hg19). For the probe design, we covered 25 bp flanking both sides of the exons to cover intron–exon boundaries.

The DNA samples of four patients were equimolarly pooled, resulting in 92 DNA pools. The DNA pools were subjected to NGS library preparation and target-enrichment according to standard protocols using a Bravo automated liquid handling platform (Agilent Technologies). NGS libraries were equimolarly pooled per 46 libraries and sequenced on a Nextseq500 sequencer (Illumina, San Diego, CA, United States) (V2, 2 × 150 bp) following the manufacturer’s instructions.

### Variant Analysis and Classification

The raw sequencing data was processed using our in-house developed pipeline, as described previously (Corsten-Janssen et al., 2020). The resulting Vcf files were uploaded into the Cartagenia/Alissa clinical informatics platform (version 5.1.4, Agilent Technologies).

First, quality filtering of the called variants was performed, excluding all those with a read depth <20 times. Next, variants with a minor allele frequency (MAF) > 0.1% in the population databases were excluded from further analysis as they are considered benign. After filtering, variants were evaluated for their potential pathogenicity using *in silico* prediction software tools that are part of the Alamut Batch software (version 2.12; Interactive Biosoftware, Rouen, France), including SIFT, PolyPhen-2, MutationTaster, AlignGVGD, PhyloP and Grantham distance and four different splice site prediction programs (NNsplice, MaxEntScan, GeneSplicer and SpliceSiteFinder-Like). Variants were classified as “likely benign” (LB), “variant of uncertain significance” (VUS), “likely pathogenic” (LP) or “pathogenic” (P), largely based on ACMG guidelines (Ellard et al., 2020). In addition, we searched scientific literature and databases that report genetic variants in patients, including the Human Genome Mutation Database (HGMD) and ClinVar, for known “P” and “LP” variants reported in SCA genes. All LP and P variants identified in our cohort have been submitted to ClinVar (September 2021). Literature was also used to verify the association of loss of function variations in exons/genes as disease mechanism for SCA.

### Pathogenicity Score Calculation and Variant of Uncertain Significance Sub-Classification

According to ACMG guidelines, *in silico* algorithm scores suggesting pathogenicity or non-pathogenicity are insufficient for clinical classification as LP, P, or LB, B, respectively. However, these scores can help in prioritizing the variants for follow-up studies. Therefore we calculated a pathogenicity score for each VUS using the scores from six independent *in silico* predictors embedded in the Alamut Batch software (SIFT, PolyPhen-2, MutationTaster, AlignGVGD, PhyloP and Grantham distance). Per variant, each predictor can have a maximum score of 1 (pathogenic) and a minimum score of 0 (non-pathogenic) (Supplementary Table S2), so variants can have a maximum score of 6 and a minimum score of 0. The VUSes were subsequently categorized into subclasses based on a cut-off of a pathogenicity score of 3, presence (MAF <0.1%)/absence in GnomAD and predicted splicing effect. Four subclasses of VUSes were identified: VUS-low: variants with a pathogenicity score <3; VUS-semi high: variants with a pathogenicity score ≥3 that were reported in GnomAD with MAF <0.1%; VUS-high: variants with a pathogenicity score ≥3 that were absent from GnomAD; and VUS-splice: variants with a predicted *in silico* splice effect when 3 or more splice site prediction programs in Alamut show a score difference between WT and variant allele.

### Confirmation of Next Generation Sequencing Detected Variants by Sanger Sequencing and Segregation Studies

Sanger sequencing was used to confirm VUS-semi high, VUS-high, VUS-splice, LP and P variants and identify the patient carrying the variant in each pool. When family members were available, Sanger sequencing was used for segregation analysis of the variant.

### Protein Modeling

We aimed to perform 3D structural modeling for VUS-semi high and VUS-high variants. Known protein structures obtained by X-ray crystallography, cryo-EM or NMR were extracted from the RCSB Protein Data Bank (rcsb.org) (Berman et al., 2000) (Table 1). For the proteins for which no experimentally determined structure was available, we performed a homology study requiring a sequence alignment of more than 40% to find suitable templates (Pearson, 2013) (Table 1). Proteins with a sequence alignment less than 40% were not included in this part of the study. SWISS-MODEL was used to identify the template that covers the region of interest and to generate a model from that template (Waterhouse et al., 2018). All models were optimized using the software Yasara Structure for loop refinement and energy minimization (Krieger and Vriend, 2015). Missense variants classified as disease-causing mutations in the HGMD database were included with the identified VUS-semi high and VUS-high variants for further modeling comparisons.

**TABLE 1:** Proteins and the associated templates used for protein modeling.

Gene	Protein	UNIPROT code	PDB access code	PDB used for homology model	Sequence alignment (%)
*AFG3L2*	Afg3l2	Q9Y4W6	6NYY		
*CACNA1A*	Cav2.1	O00555		5GJW	49
*DAB1*	Dab1	O75553	1NTV		
*PRKCG*	PKCγ	P05129	2UZP		
*SPTBN2*	Sptbn2	O15020		1SJJ	47
*TGM6*	Tgm6	O95932		3S3S	42
*PDYN*	Pdyn	P01213	2N2F		

### Cell Culture and Transfection

HEK293T and SH-SY5Y cells were selected for functional studies. The HEK293T cell line is commonly used to assess splice effects of DNA variants with a minigene assay and has previously been used to study the functional consequence of SCA variants (Nibbeling et al., 2017). SH-SY5Y cells are derived from human neuroblastoma cells and are widely used as a model for neurodegenerative disorders (Schlachetzki et al., 2013). Cells were grown in Dulbecco’s modified Eagle medium supplemented with 10% fetal bovine serum and 1% penicillin-streptomycin in a 37°C incubator with 5% CO_2_. Transfections were performed using polyethylenimine (Polysciences, PA, United States) according to the manufacturer’s instructions. Approximately 50,000 cells were grown on uncoated glass coverslips in 24-well plates for immunocytochemistry and in 6-well plates for the Minigene splicing assay. Cells were cultured 48 h post-transfection.

### Minigene Splicing Assay

The splicing reporter Minigene assay was used to investigate the implication of VUS-splice variants for splicing of the corresponding mRNA transcripts. Conform to our standard diagnostic procedure, variants occurring in ±3–20 bp of exon-intron boundaries and predicted to have an effect on splicing by at least 3 of the 4 Alamut Bench software splice prediction programs were selected. To predict functional consequences of these variants suspected of having a splice effect, the Human Splicing Finder 3.1 program was used as previously described (Desmet et al., 2009). The program generates Consensus Values (CV) in a range from 0 to 100 for each nucleotide input. Wildtype and Mutant sequences were uploaded in the program and differences between the Consensus values were analyzed. This combined analysis prioritized variants that most likely create a new donor or acceptor site that would be used as an alternative donor or acceptor *in vivo*, for functional follow-up studies. In brief, the region of interest in the genomic DNA of the patient was PCR-amplified and then cloned into the Minigene pSPL3 exon-trapping vector (Thermo Fisher Scientific, MA, United States). After 48 h of transfection, total RNA was isolated from HEK293T or SH-SY5Y cells expressing either a control (empty pSPL3 vector) or wild type pSPL3 vector (containing the fragment of interest without the VUS-splice) or a mutant pSPL3 vector (containing the fragment of interest with the VUS-splice) with TRIzol (Invitrogen, CA, United States). RNA was converted to cDNA using the RevertAid H Minus First Strand cDNA Synthesis Kit, according to the manufacturer’s protocol (Thermo Fisher Scientific). The cDNA was PCR-amplified using specific primers (Supplementary Table S3), and the PCR fragments were analyzed by agarose gel electrophoresis. The PCR products were Sanger sequenced to reveal the exact effect on splicing.

### DNA Plasmids

For the translocation and immunochemistry experiments, we used previously described constructs encoding for wild type PKCγ-EGFP and wild type TGM6-Myc (Verbeek et al., 2008; Nibbeling et al., 2017). The variants in the respective genes were introduced using the QuickChange II XL Site-Directed Mutagenesis Kit (Agilent Technologies) following the manufacturer’s protocol (Supplementary Table S3). The plasmid sequences were verified by Sanger sequencing.

### Immunocytochemistry

HEK293T cells expressing wild type and mutant PKCγ were treated with PMA (20 nM, 10 min) (Sigma Aldrich, St. Louis, MO, United States) to obtain PKCγ translocation. Cells were fixed with 4% paraformaldehyde in phosphate-buffered saline (PBS) for 15 min at room temperature and washed three times with PBS. With the exception of PKCγ-EGFP transfected cells, cells were permeabilized and blocked in 0.1% Triton™ X-100, 5% fetal bovine serum in PBS for 30 min at room temperature, followed by incubation overnight at 4°C with primary antibody mouse anti-Myc (Cell Signaling, Danvers, MA, United States; 1:100). The next day, the cells were washed with PBS and incubated with secondary antibody including mouse anti-Alexa Fluor® 488 (Santa Cruz Biotechnology, TX, United States; both 1:500) for 1 h at room temperature in phosphate buffer. All slides were mounted in Vectashield medium with 4′,6-diamidino-2-phenylindole (DAPI; Vector Laboratories, CA, United States). Images were obtained with structured illumination microscopy and processed using ImageJ software (http://fiji.sc/, National Institutes of Health, Bethesda, MD, United States).

## Results

### Routine Genetic Diagnostic Classification of Variants

After duplicate read removal, we obtained 375 Kb unique reads on average per patient, resulting in 25x coverage for 90% of the bp per individual patient. The sequencing of one pool failed and four pools had too low coverage, and these pools were excluded from the study, leaving 348 patients (out of 368) for further analysis.

**FIGURE 1:**
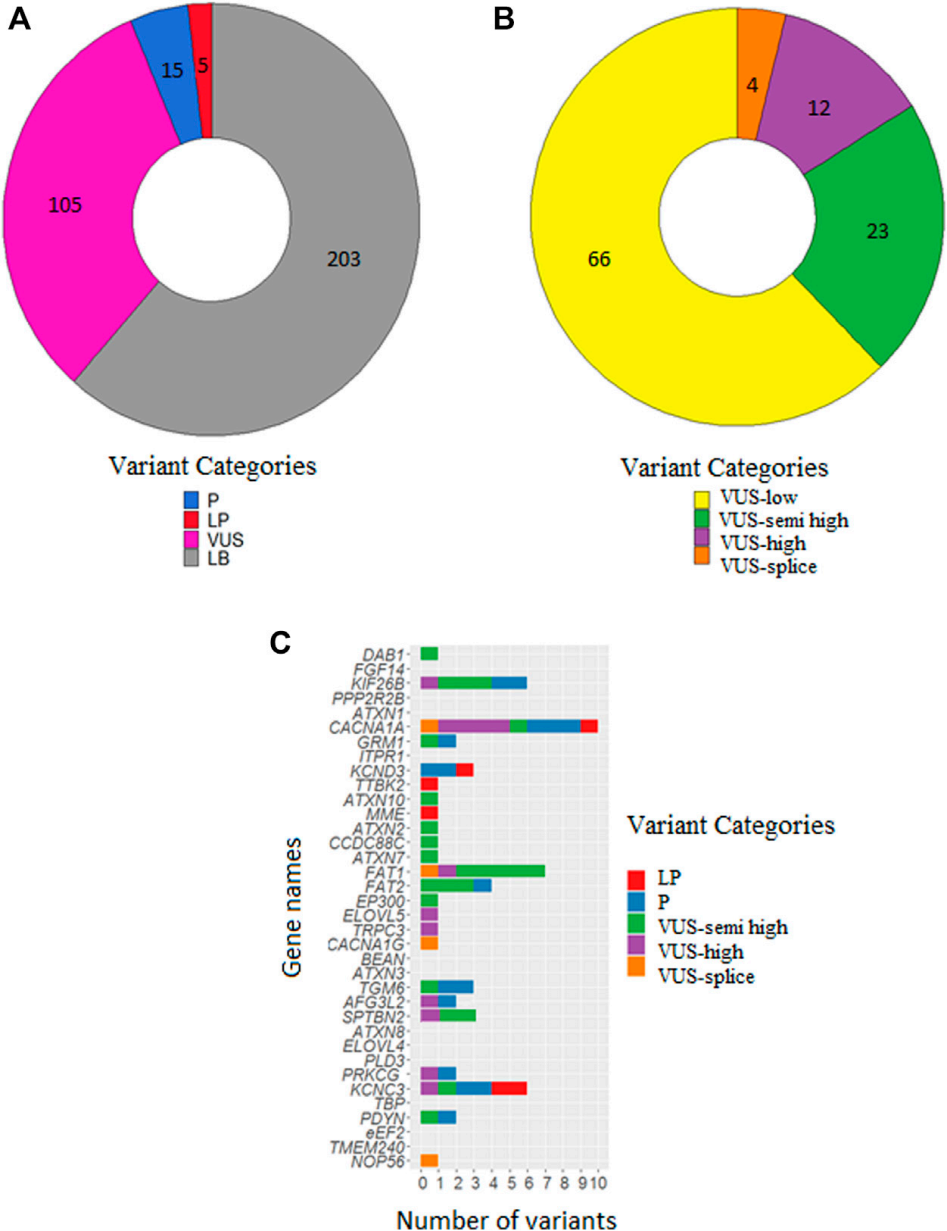
Numbers of classified variants in 348 SCA patients. **(A)** Number of variants classified as P, LP, VUS and LB. **(B)** Number of VUSes classified as VUS-low, VUS-semi high, VUS-high and VUS-splice. **(C)** Distribution of P/LP variants, VUS-semi high, VUS-high and VUS-splice over the 36 SCA genes. The list is sorted on gene size with the largest on top (*DAB1*: 1,551,957 bases) and the smallest on bottom (*NOP56*: 5,801 bases).

After filtering, 328 unique variants were identified in the 348 patients. Based on our in-house routine diagnostic tree, 15 variants were classified as P, 5 variants as LP, 105 variants as VUS and 203 variants as LB (Figure 1A). Of the 20 LP/P variants, 5 had not previously been reported in the literature. These 20 LP/P variants provided a genetic diagnosis for 22 patients (6.3%) and were located in 12 different SCA genes, with the most prevalent genes being *KCNC3/*SCA13 (4 diagnoses), *CACNA1A/*SCA6 (4 diagnoses), *KCND3/*SCA19/22 (3 diagnoses) and 9 other SCA genes in which LP/P variants were identified (one or two diagnoses each) (Table 2).

**TABLE 2:** Pathogenic (P) and likely pathogenic (LP) variants identified in 22 patients.

Patient ID	Gene name	SCA type	Ref seq number	cDNA: protein position	Class	References
1	*CACNA1A*	SCA6	NM_023035.2	c.835C>T: p.R279C	P	Maksemous et al. (2016)
2	*CACNA1A*	SCA6	NM_023035.2	c.1748G>A: p.R583Q	P	Battistini et al. (1999)
3	*CACNA1A*	SCA6	NM_023035.2	c.3426delG: p.T1143Pfs*47	P	—
4	*CACNA1A*	SCA6	NM_023035.2	c.4997G>A: p.R1666H	LP	Tantsis et al. (2016)
5	*TTBK2*	SCA11	NM_173500.3	c.3466C>T: p.R1156*	LP	—
6, 7	*KCNC3*	SCA13	NM_004977.2	c.1771A>G: p.S591G	LP	Duarri et al. (2015)
8	*KCNC3*	SCA13	NM_004977.2	c.1268G>A: p.R423H	P	Figueroa et al. (2010)
9	*KCNC3*	SCA13	NM_004977.2	c.1259G>A: p.R420H	P	Waters et al. (2006)
10	*PRKCG*	SCA14	NM_001316329.1	c.107A>G: p.H36R	P	Németh et al. (2013)
11	*KCND3*	SCA19/22	NM_004980.4	c.1054A>C: p.T352P	P	Duarri et al. (2012)
12	*KCND3*	SCA19/22	NM_004980.4	c.1169G>A: p.S390N	P	Duarri et al. (2012)
13	*KCND3*	SCA19/22	NM_004980.4	c.1291C>T: p.R431C	LP	Choi et al. (2017)
14	*PDYN*	SCA23	NM_001190892.1	c.632T>C: p.L211S	P	Bakalkin et al. (2010)
15	*AFG3L2*	SCA28	NM_006796.2	c.1861C>G: p.L621V	P	Nibbeling et al. (2017)
16	*TGM6*	SCA35	NM_198994.2	c.691C>T: p.R231*	P	—
17	*TGM6*	SCA35	NM_198994.2	c.1429_1430insTCTCT: p.G477Vfs*28	P	—
18	*MME*	SCA43	NM_000902.3	c.1095-2A>C	LP	—
19	*GRM1*	SCA44	NM_001278064.1	c.2375A>G: p.Y792C	P	Watson et al. (2017)
20	*FAT2*	SCA45	NM_001447.2	c.10758G>C: p.K3586N	P	Nibbeling et al. (2017)
21^a^, 22^B^	*KIF26B*	—	NM_018012.3	c.5710G>A: p.D1904N	P	Nibbeling et al. (2017)

a Marker analysis showed no evidence of a shared ancestor with patient 22.

b Marker analysis showed no evidence of a shared ancestor with patient 21.

### Prioritization of VUSes for Further Follow-Up

Following diagnostic classification, as described in the *Materials and Methods*, we subclassified the VUSes to prioritize them for follow-up studies. The 66 VUS-low variants (pathogenicity score <3) were excluded from further follow-up. We then selected 23 VUS-semi high (pathogenicity score ≥3 and MAF <0.1% in GnomAD), 12 VUS-high (pathogenicity score ≥3 and absent in GnomAD) and 4 VUS-splice (predicted splice effect) for putative follow-up studies (Figure 1B; Table 3). In total, 39 VUS-semi high, VUS-high and VUS-splice were identified in 37 patients, with 3 patients carrying two VUSes in different genes. One VUS-semi high in *KIF26B* was detected in two seemingly unrelated patients (numbers 25, 56). Two patients carried a known P/LP variant and a VUS-semi high: patient 9 carried a P variant in *KCNC3* (c.1259G>A: p.R420H) and a VUS-semi high in *TGM6* (c.1171G>A: p.V391M), and patient 4 carried an LP variant in *CACNA1A* (c.4997G>A: p.R1666H) and a VUS-semi high in *PDYN* (c.635G>A: p.R212Q) (Tables 2, 3). The P variants, LP variants, VUS-semi high, VUS-high and VUS-splice were distributed across 24 genes, with *CACNA1A* carrying the highest number of variants (n = 10; Figure 1C), an observation that is not completely attributable to the size of *CACNA1A*, as the much larger *DAB1* gene only carried one variant (Figure 1C).

**TABLE 3:** List of VUS-semi high, VUS-high and VUS-splice identified in 36 patients.

Patient ID	Gene name	SCA type	Refseq number	cDNA: Protein position	Pathogenicity score^a^	GnomAD allele frequency	Subclass
23	*ATXN2*	SCA2	NM_002973.3	c.974T>C: p.M325T^1^	3	1/245828	VUS-semi high
24	*SPTBN2*	SCA5	NM_006946.3	c.7109G>A: p.R2370H	3.2	24/245754	VUS-semi high
25	*SPTBN2*	SCA5	NM_006946.3	c.6169G>T: p.A2057S	4	—	VUS-high
	*KIF26B*	—	NM_018012.4	c.2605G>A: p.G869R	4.1	12/276886	VUS-semi high
26	*SPTBN2*	SCA5	NM_006946.3	c.1522A>C: p.N508H	5	17/272418	VUS-semi high
27	*CACNA1A*	SCA6	NM_023035.2	c.6418C>T: p.R2140C	3.1	6/96904	VUS-semi high
	*FAT1*	—	NM_005245.3	c.8991G>A	—	—	VUS-splice
28	*CACNA1A*	SCA6	NM_023035.2	c.5669T>A: p.V1890D	3.1	—	VUS-high
4	*PDYN*	SCA23	NM_024411.4	c.635G>A: p.R212Q	4.1	26/282806	VUS-semi high
29	*CACNA1A*	SCA6	NM_023035.2	c.3161T>C: p.I1054T	3	—	VUS-high
30	*CACNA1A*	SCA6	NM_023035.2	c.2357G>C: p.R786P	3.1	—	VUS-high
31	*CACNA1A*	SCA6	NM_023035.2	c.1586T>C: p.L529P	3	—	VUS-high
32	*CACNA1A*	SCA6	NM_023035.2	c.5157T>A	—	—	VUS-splice
33	*ATXN7*	SCA7	NM_000333.3	c.2528C>T: p.S843L	3.1	9/246040	VUS-semi high
34	*ATXN10*	SCA10	NM_013236.3	c.404G>T: p.G135V	3.1	50/277128	VUS-semi high
35	*KCNC3*	SCA13	NM_004977.2	c.1130T>C: p.L377P	4.1	—	VUS-high
36	*KCNC3*	SCA13	NM_004977.2	c.1876G>T: p.G626W	4	2/131410	VUS-semi high
37	*PRKCG*	SCA14	NM_002739.3	c.715C>T: p.R239W	3.2	—	VUS-high
38	*AFG3L2*	SCA28	NM_006796.2	c.2143C>T: p.L715F	4	—	VUS-high
	*TRPC3*	SCA41	NM_001130698.1	c.949G>A: p.E317K	3.2	—	VUS-high
9	*TGM6*	SCA35	NM_198994.2	c.1171G>A: p.V391M	4	206/277194	VUS-semi high
39	*NOP56*	SCA36	NM_006392.3	c.909G>A	—	—	VUS-splice
40	*DAB1*	SCA37	NM_021080.4	c.209G>A: p.G70D	3	1/223040	VUS-semi high
41	*ELOVL5*	SCA38	NM_001301856.1	c.490G>A: p.G164S	3	—	VUS-high
42	*CCDC88C*	SCA40	NM_001080414.3	c.6026C>T: p.P2009L	4	168/270454	VUS-semi high
43	*CACNA1G*	SCA42	NM_018896.4	c.3792G>T	—	—	VUS-splice
44	*GRM1*	SCA44	NM_001278064.1	c.3236C>T: p.P1079L	3	1/243296	VUS-semi high
45	*FAT2*	SCA45	NM_001447.2	c.12899T>C: p.M4300T^2^	3	2/30892	VUS-semi high
46, 47	*FAT2*	SCA45	NM_001447.2	c.12464C>G: p.S4155C^3^	3.2	231/274944	VUS-semi high
48	*EP300*	—	NM_001429.3	c.214C>A: p.Q72K	3.2	7/246266	VUS-semi high
49	*FAT1*	—	NM_005245.3	c.8041C>T: p.P2681S	5	11/277060	VUS-semi high
50	*FAT1*	—	NM_005245.3	c.7130C>T: p.T2377M	3	186/277024	VUS-semi high
51	*FAT1*	—	NM_005245.3	c.6808G>A: p.D2270N	4	12/276942	VUS-semi high
52	*FAT1*	—	NM_005245.3	c.5762G>T: p.G1921V	5.1	—	VUS-high
53	*FAT1*	—	NM_005245.3	c.1940C>T: p.A647V	5	23/277126	VUS-semi high
54	*FAT1*	—	NM_005245.3	c.544G>A: p.G182R	5.1	8/277210	VUS-semi high
55	*KIF26B*	—	NM_018012.4	c.1340G>A: p.G447E	4	—	VUS-high
56	*KIF26B*	—	NM_018012.4	c.2605G>A: p.G869R	4.1	12/276886	VUS-semi high
57	*KIF26B*	—	NM_018012.4	c.2023G>C: p.D675H	4	10/245992	VUS-semi high

a Since the pathogenicity score is not calculated for VUS-splice it is displayed as “-”.

1 The VUS was excluded from the re-classification because the patient carrying the VUS received a diagnosis for autosomal recessive spinocerebellar ataxia-17.

2 The VUS was excluded from the re-classification because the patient carrying the VUS received a diagnosis for multiple system atrophy.

3 The VUS was excluded from the re-classification because the patient carrying the VUS received a diagnosis for epilepsy.

### Follow-Up Studies After Routine Genetic Diagnostics to Reclassify VUSes

When possible, we performed follow-up studies to reclassify VUS-semi high, VUS-high and VUS-splice, including segregation studies, protein modeling and functional tests. In total, it was possible to perform at least one follow-up study for 13 VUS-semi high, VUS-high and VUS-splice (Table 4).

**TABLE 4:** Reclassification of VUSes based on follow-up studies.

Patient ID	Gene	cDNA: Protein position	Subclass	Follow-up studies	Follow-up outcome	Final classification
38	*AFG3L2*	c.2143C>T: p.L715F	VUS-high	PM	PM predicted LB	VUS
28	*CACNA1A*	c.5669T>A: p.V1890D	VUS-high	PM	PM predicted LP	VUS
30	*CACNA1A*	c.2357G>C: p.R786P	VUS-high	SS	SS indicates LP	LP
31	*CACNA1A*	c.1586T>C: p.L529P	VUS-high	PM	PM predicted LP	VUS
32	*CACNA1A*	c.5157T>A	VUS-splice	FT	FT indicates LB	VUS
43	*CACNA1G*	c.3792G>T	VUS-splice	FT	FT indicates LP	LP
40	*DAB1*	c.209G>A: p.G70D	VUS-semi high	PM	PM predicted LB	VUS
27	*FAT1*	c.8991G>A	VUS-splice	FT	FT indicates LB	VUS
39	*NOP56*	c.909G>A	VUS-splice	FT	FT indicates LP	LP
4	*PDYN*	c.635G>A: p.R212Q	VUS-semi high	PM	PM predicted LP, segregates with known LP in CACNA1A	VUS
37	*PRKCG*	c.715C>T: p.R239W	VUS-high	PM, SS, FT	PM predicted LP, SS and FT indicate LP	LP
26	*SPTBN2*	c.1522A>C: p.N508H	VUS-semi high	PM	PM predicted LB	VUS
9	*TGM6*	c.1171G>A: p.V391M	VUS-semi high	PM, FT	PM predicted LB, FT indicates LB, segregates with known P variant in *KCNC3*	VUS

LB, likely benign; LP, likely pathogenic; PM, protein modeling; SS, segregation studies; FT, functional test.

### Segregation Studies

For the 39 VUSes (VUS-semi high, VUS-high and VUS-splice), family members were available for only two patients carrying a VUS-high (Table 4): the affected father and grandfather of patient 30 both carried the c.2357G>C: p.R786P variant in *CACNA1A* and the affected mother of patient 37 with c.715C>T: p.R239W in *PRKCG* also carried this variant. Both VUS-high thus segregated with the disease in the family, which supports them being LP variants.

### Protein Modeling

Since the corresponding protein structure was not available for all VUS-semi high and VUS-high, we could only perform protein modeling for four VUS-semi high and four VUS-high (Table 4). Below we discuss the impact of the four VUSes for which protein modeling indicated a potentially pathogenic change in structure.

#### R239W VUS-High in the PKCγ Protein

**FIGURE 2:**
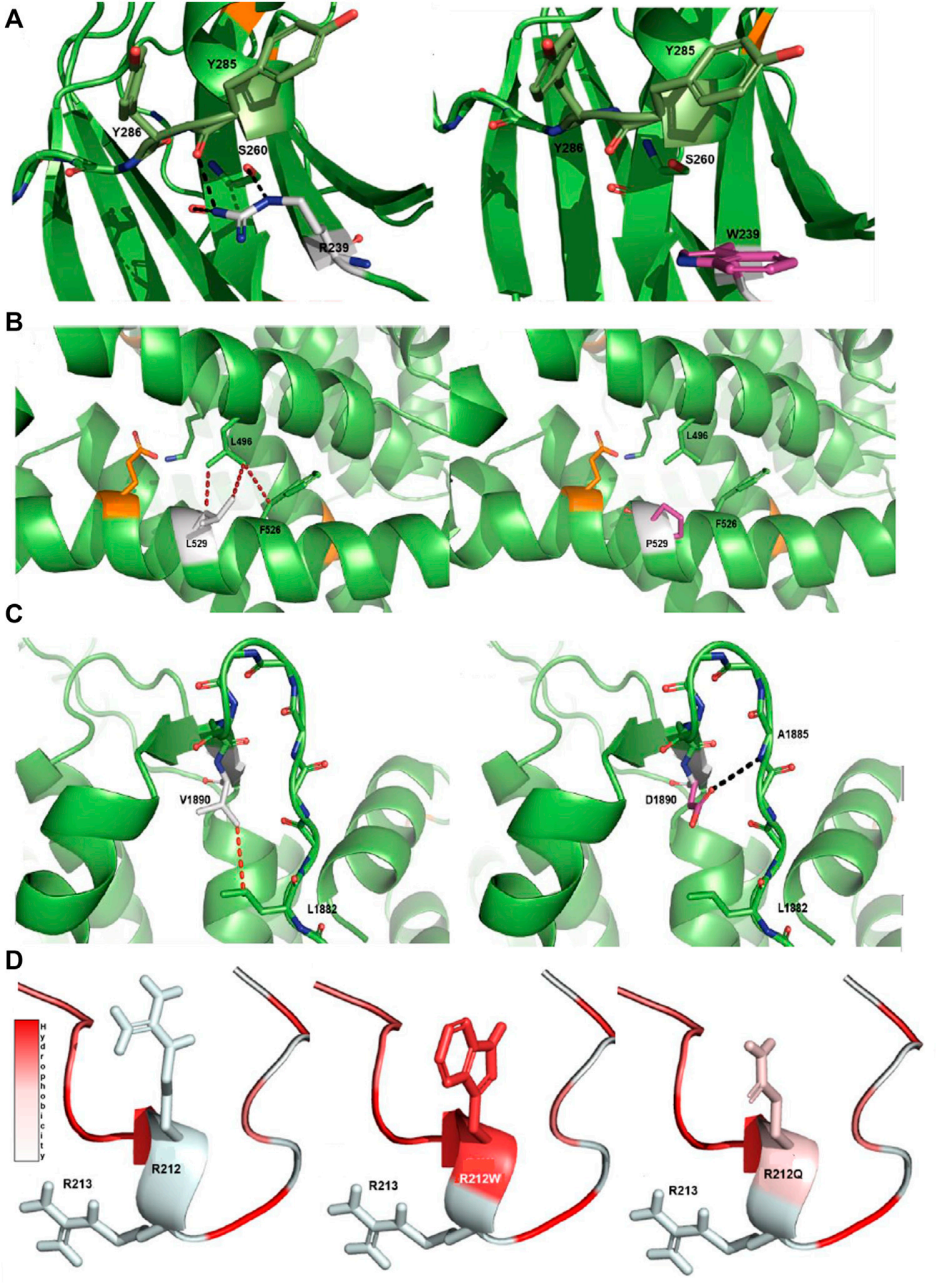
Protein modeling of variants with a notable effect on protein structure. **(A)** R239W VUS-high in PKCγ protein. The native R239 interacts with S260 and Y285 through a hydrogen bonding network. The aromatic side chain of the mutant W is unable to establish an interaction network in the same manner as the native arginine. **(B)** L529P VUS-high in Cav2.1 protein. The native L529 engages in a network of hydrophobic interactions with L496 and F526. The introduction of P in the middle of the helix introduces a steric clash with these hydrophobic residues and is detrimental for helix stability (Visiers et al., 2000). **(C)** V1890D VUS-high in Cav2.1 protein. The native V1890 interacts with L1882 through a hydrophobic interaction. The mutated D changes the conformation of the backbone of the protein, disturbing the β-sheet structure and forming a new hydrogen bond with the main chain of A1885. Native side chains are shown as white, mutated side chains as pink, known pathogenic mutations as orange, hydrophobic interactions as red dashes and hydrogen bonds as black dashes. **(D)** R212Q VUS-semi high in pdyn protein. The R212Q mutation has similar hydrophobic properties to the known R212W, which is known to decrease the cleavage efficiency of dynorphin, and likely functions in a similar manner. Arginine (R), tryptophan (W), serine (S), tyrosine (Y), valine (V), aspartic acid (D), leucine (L), proline (P), glutamine (Q) and phenylalanine (F).

The substitution of an arginine (R) at position 239 to a tryptophan (W) in PKCγ (*PRKCG*) is predicted to affect the protein structure. The native R at position 239 establishes a network with serine (S) at 260 and tyrosine (Y) at 285. The introduction of a W at this position is a non-conservative replacement of the positive charged R with an uncharged aromatic W, which cannot establish a network similar to that associated with the native R (Figure 2A).

#### L529P VUS-High in Cav2.1 Protein

The substitution of a leucine (L) at position 529 with a proline (P) in Cav2.1 (*CACNA1A*) is very likely detrimental for the protein structure. The native L at position 529 interacts with L496 and a phenylalanine (F) at 526 through hydrophobic interactions. The introduction of a P in the middle of an α-helix crashes these hydrophobic interactions, which is most likely detrimental for the helix stability and may also lead to increased flexibility of the helix (Figure 2B) (Visiers et al., 2000).

#### V1890D VUS-High in Cav2.1 Protein

The substitution of a valine (V) at position 1890 with an aspartic acid (D) most likely has a substantial impact on the Cav2.1 protein. The V at position 1890 interacts with L1882 through a hydrophobic interaction. The introduction of the charged D at this position shifts the network of interactions within the backbone of the protein. The torsion of the charged side chain disturbs the β-sheet structure through its rotation and forms a new hydrogen bond with the backbone of alanine (A) at 1885, indicating that this variant has an impact on the protein structure (Figure 2C).

#### R212Q VUS-Semi High in Pdyn Protein

The substitution of R to glutamine (Q) at position 212 can have effects on the cleavage of dynorphin (*PDYN*). Previous studies report R212W as damaging, with lower levels of cleavage of dynorphin into smaller peptides (Bakalkin et al., 2010; Madani et al., 2011). Based on the changes in hydrophobicity, R212Q can also decrease the cleavage efficiency of dynorphin into smaller peptides, similar to what happens with R212W (Figure 2D).

The remaining three VUS-semi high (*DAB1* c.209G>A: p.G70D, *SPTBN2* c.1522A>C: p.N508H and *TGM6* c.1171G>A: p.V391M) and one VUS-high (*AFG3L2* c.2143C>T: p.L715F) are seemingly not detrimental for helix stability or β-sheet structure and/or are not located in functionally relevant areas of the protein structure (Supplementary Figures S1–S4; Supplementary Results).

In summary, we identified three VUS-high and one VUS-semi high that seem to be deleterious for the corresponding protein structures and support a LP classification.

### Cellular Studies

**FIGURE 3:**
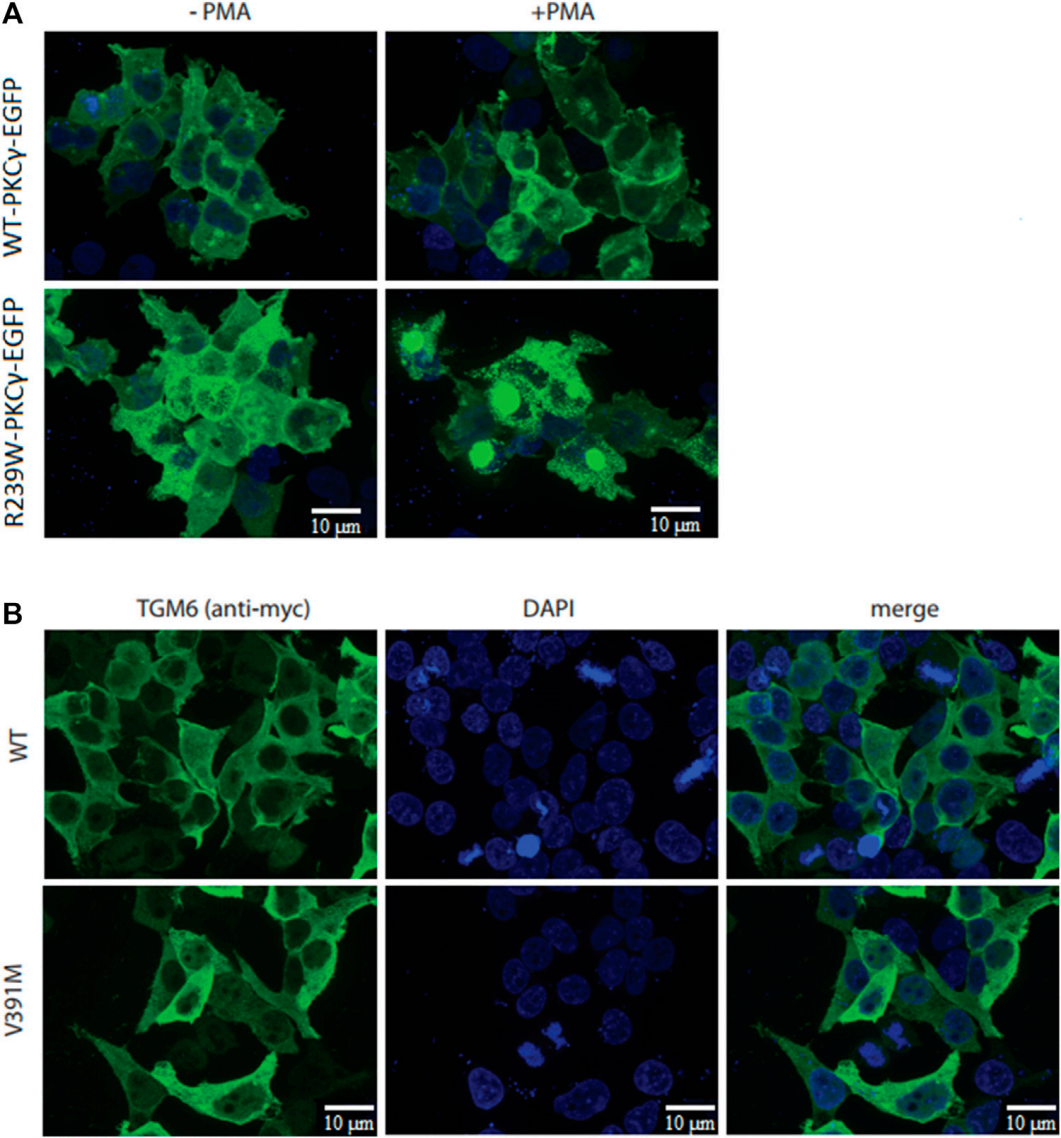
Cellular studies of R239W PKCγ and V391M TGM6. **(A)** While PMA-induced translocation was observed for wild type PKCγ-EGFP, very little translocation was observed for R239W-PKCγ-EGFP. **(B)** Immunocytochemistry did not show any difference in protein localization between wild type TGM6 and V391M-TGM6.

Functional assays were operational for the p.R239W VUS-high in the PKCγ protein and p.V391M VUS-semi high in the TGM6 protein. The p.R239W VUS-high located in the catalytic domain of PKCγ protein (patient 37; Table 4) was analyzed by determining its cellular localization and response to phorbol ester–induced plasma membrane translocation, as this was previously reported to be altered by numerous SCA14 mutations (Seki et al., 2005; Verbeek et al., 2008). We transiently transfected HEK293T cells with either wild type PKCγ-EGFP or R239W-mutant PKCγ-EGFP following 10 min of phorbol 12-myrisate 13-acetate (PMA) treatment. Whereas we observed mild PMA-induced translocation for wild type PKCγ-EGFP, this was barely seen for R239W PKCγ-EGFP due to cytoplasmic protein aggregation that we could already observe in the untreated R239W PKCγ-EGFP–expressing cells. Protein aggregation was not observed for wild type PKCγ-EGFP using fluorescent microscopy (Figure 3A). Intracellular protein aggregation was previously reported for PKCγ proteins carrying known pathogenic missense variants (Seki et al., 2005).

In addition, to study the functional effects of the p.V391M VUS-semi high variant in TGM6 (patient 9, who also carried a known pathogenic variant in *KCNC3*, Table 4), we determined the protein localization of wild type TGM6 and V391M-TGM6 in transiently transfected HEK293T cells as cellular localization was reported to be linked with pathogenic missense variants (Nibbeling et al., 2017). However, the immunocytochemistry did not show any difference in protein localization in the cytoplasm between wild type TGM6 and V391M-TGM6 (Figure 3B), which supports a likely benign classification.

### Minigene Splicing Assays

Alamut Bench software predicted 17 variants to have a potential effect on splicing (Supplementary Table S4), i.e., >3 algorithms predicted the emergence or (almost) loss of a donor or acceptor site, according to our diagnostic criteria. After carefully verifying the prediction outcome, four variants in *CACNA1G* (c.3792G>T)*, CACNA1A* (c.5157T>A), *FAT1* (c.8991G>A) and *NOP56* (c.909G>A) were considered VUS-splice and were followed up by a Minigene splicing assay to verify the *in silico* predicted splicing effects.

**FIGURE 4:**
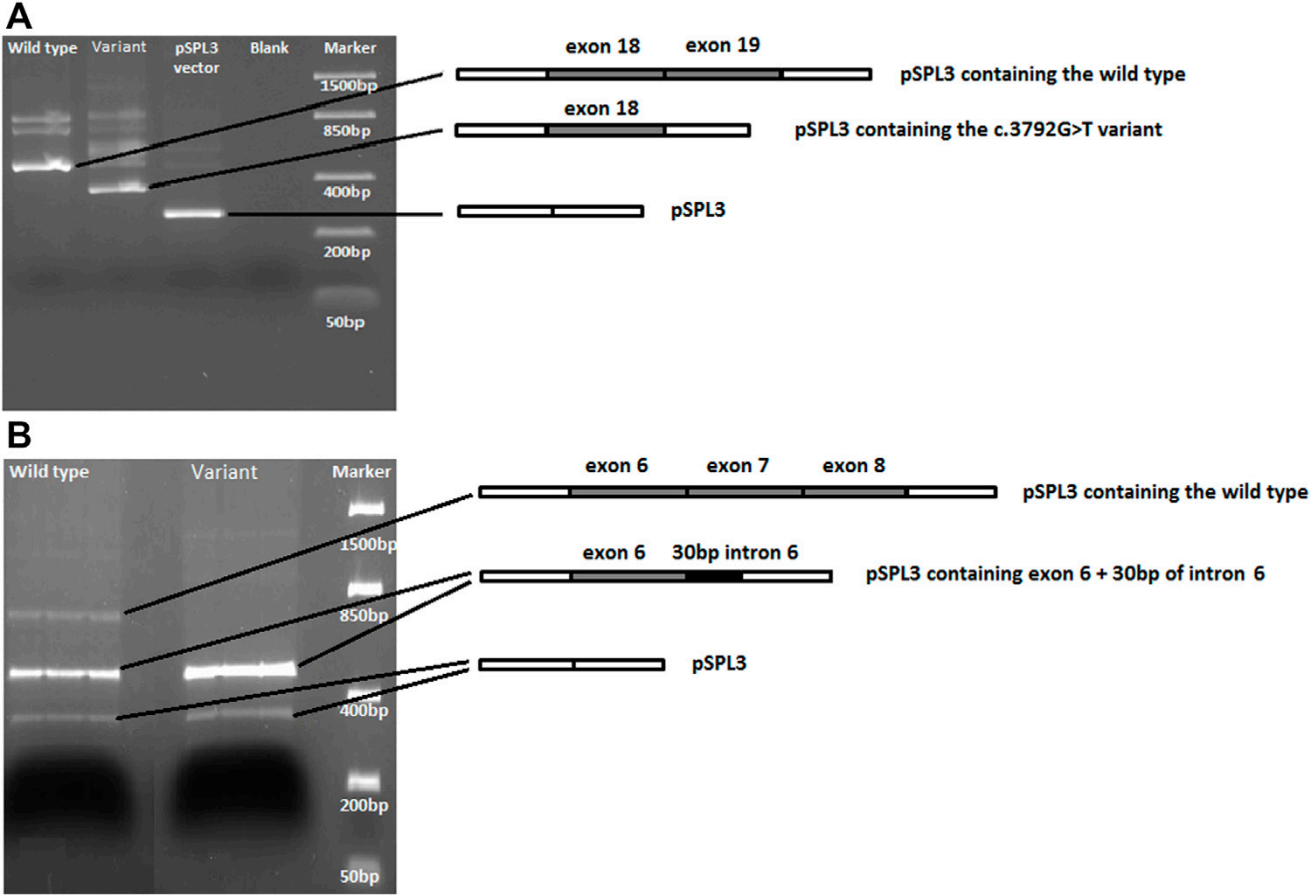
DNA gel electrophoresis of PCR-amplified cDNA fragments generated from wild type and variant sequences. **(A)** For the *CACNA1G* c.3792G>T variant, the variant fragment is shorter and lacks exon 19 compared to the wild type fragment. **(B)** For *NOP56*, both the wild type and the c.909G>A variant sequences produce a fragment carrying exon 6 and 30bp of intron 6. However, the c.909G>A variant PCR product lacks the expected cDNA fragment carrying exons 6, 7 and 8.

For the c.3792G>T VUS-splice, the first nucleotide of exon 19 of *CACNA1G,* Alamut predicted a weakened original splice acceptor site. This was confirmed by Minigene splicing assay as the variant transcript produced a shorter cDNA fragment (364 bp) compared to the fragment (488 bp) generated from the wild type transcript (Figure 4A), and this coincided with a complete loss of exon 19 (124 bases) shown by Sanger sequencing. These results confirm that c.3792G>T VUS-splice weakens the original acceptor site and uses the acceptor site of exon 20, leading to complete skipping of exon 19 and, consequently, to a frameshift and putative truncating Cav3.1 protein.

For c.909G>A VUS-splice, located at the end of exon 7 of *NOP56*, Alamut predicted a loss of the original splice site. The Minigene splicing assay results showed that, while the wild type transcript did produce the expected cDNA fragment carrying exons 6, 7, and 8, this fragment was not generated by the variant transcript. Interestingly, a cDNA fragment carrying exon 6 and 30 bp of intron 6 was generated from both the wild type and variant transcripts (Figure 4B). These results show that the c.909G>A that causes the loss of the original splice site most likely leads to use of a splice site in the downstream exons and consequently to a putative truncating NOP56 protein.

The Minigene assays of the other two VUS-splice (*CACNA1A*; c.5157T>A and *FAT1*; c.8991G>A) showed no difference in splicing between the wild type and variant transcripts (Supplementary Figure S5).

### Variant of Uncertain Significance Re-Classification

The results of the follow-up studies were discussed with a laboratory specialist (HL) and a clinical geneticist (CVB), leading to the re-classification of two VUS-high and two VUS-splice to LP variants (Table 4).

## Discussion

After screening 36 SCA genes in 348 patients with cerebellar ataxia, 6.3% of cases received a genetic diagnosis (carrying LP or P variants) and 93.7% carried a VUS and/or LB variant using routine diagnostic criteria. Using follow-up studies, we were able to reclassify 4 of the 13 VUSes (30%) to LP, increasing our diagnostic yield to 7.4%. The diagnostic yields in previous studies that used panel or exome sequencing on undiagnosed cerebellar ataxia patients were between 7.2% and 21% (Németh et al., 2013; Fogel et al., 2014; Coutelier et al., 2017). However, some variants reported in these studies were transmitted in a recessive mode, which was not covered by our study, and this difference can explain our relatively low diagnostic yield compared to these studies. *CACNA1A* carried the most pathogenic variants and VUSes, as also reported by others (Coutelier et al., 2017), and is thus one of the most prevalent SCA genes, carrying both polyQ repeat expansions and conventional variations.

After prioritizing VUSes for follow-up studies using our VUS classification system, we were able to investigate 13 out of 39 prioritized variants (23 VUS-semi high, 12 VUS-high and 4 VUS-splice) using variant segregation studies, protein modeling and cellular studies. We could re-classify two VUS-high to LP variants. For the four VUS-splice variants for which we tested the predicted splice effect, we could reclassify two to LP based on the outcome of the splicing assay.

In its current form the ACMG rules can use evidence from functional analysis, but much weight is carried by “traditional” types of clinical evidence like segregation studies and population frequencies. The major challenge to re-classify VUSes is the lack of availability of DNA material from other affected family members for segregation studies. Collecting other affected family members is challenging for a late-onset disease like SCA, and even when family members are available, families are often too small to yield sufficient evidence. In our 13 patients with a VUS, other affected family members were available for only two patients. In these two cases, variant segregation analysis enabled us to re-classify these two VUS-high to LP, demonstrating again that engaging patients and their family members remains the most clear-cut solution for VUS re-classification. Still, even given the best efforts, in our experience in genetics diagnostics, availability of bio specimens from enough affected relatives, preferably distant from each other in the pedigree, to reach convincing statistical evidence is difficult. It would be a major boost to variant classification if functional analysis and evidence from *in silico* analysis including protein modeling would be relatively simple to operate and reliably correlate with risk of clinical disease. Examples of such high predictability from functional analysis have already been published (Drost et al., 2019). Obviously, providing such evidence for clinical risks based on non-clinical/epidemiological data is a big challenge and may not be realistic for rare disorders where such evidence would be more difficult to collect.

In this study, 3D protein modeling analyses were possible for 8 of the 35 VUS-semi high and VUS-high. Two VUS-high in *CACNA1A*, one VUS-high in *PRKCG* and one VUS-semi high in *PDYN* were predicted to have a detrimental effect on the structure of the protein via disruption of the native conformation. However, whether those disruptions lead to a change in protein function/activity remains unknown, with the exception of the VUS-high in PKCγ which showed an impaired phorbol-induced plasma membrane translocation and protein aggregation. According to the ACMG guidelines, these four variants will still be classified as VUS, but the introduction of 3D protein modeling helps to select VUSes worth following up with additional functional studies.

In our view, more protein modeling experts are needed in the genome diagnostics setting to interpret the effects of VUSes on proteins. Additionally, genetic and protein modeling data-sharing across centers is essential to evaluate modeling data for every individual protein or even protein domain. Modeling data for numerous (L)B and (L)P variants present in the same protein domain structure and/or affecting the protein structure in a similar manner should be collected with data confirming its pathogenicity such as functional experiments or segregation analysis. So, for now protein modeling is a tool that gives additional insight in the pathogenicity of the variants and can only be used as a prediction tool for further follow up studies. Ultimately, for some proteins or protein domains, we will gain enough knowledge to increase the weight of protein modeling outcomes in reclassification strategies without the need of using functional data.

In this study, four prioritized VUS-splice were functionally tested in a Minigene splicing assay. Of these, only the *CACNA1G*; c. 3792G>T and *NOP56*; c.909G>A variants, both located at the intron/exon boundaries, showed a splicing effect concordant with the *in-silico* predictions. The remaining two VUS-splice, which are located in the middle of the exon, were *in-silico* predicted to be potentially splice-altering, but this was not confirmed with the Minigene assay. The discrepancy between the prediction and the *in vitro* test may reflect the fact that splice effect–predicting algorithms do not perform particularly well for variants located in the middle of exons (Anna and Monika, 2018). In contrast, even though the Minigene splicing reporter assay is a reliable method, the outcome of this *in vitro* assay may not be the same as that occurring in the *in vivo* tissue of interest, the cerebellum. This is reinforced by our finding that the *NOP56*; c.909G>A variant only showed a splicing effect in neuroblastoma SHSY5Ycells. We therefore strongly encourage genome diagnostic centers to either perform Minigene assays to assess the functional impact on splicing of variants other than consensus splice site variants, or to use RT-PCR in a disease-relevant cell type, or to perform RNA-seq to reveal splice effects. This will not only increase genetic diagnostic yield, it will also help optimize artificial intelligence–based prediction algorithms like spliceAI and SQUIRLS (Jaganathan et al., 2019; Danis et al., 2021).

In conclusion, our study shows that reclassification of VUSes is difficult due to the limited opportunities to carry out variant segregation studies and the limited weight and availability of protein modeling and functional tests. We anticipate that this situation will not change on the near future because the AMCG classification guidelines are very stringent, diagnostic labs lack protein modeling experts and functional tests are not easily available. Thus, we advise focusing first on the most clinically relevant genes, gaining expertise on the structure of these proteins, bringing trained protein modeling experts into diagnostics and making functional testing part of routine diagnostics. To improve this yield further, VUSes and available clinical, functional and protein modeling data should be shared more widely between labs in order to build sufficient knowledge to better weigh the value of those outcomes for classification and thereby give them a more prominent position in classification guidelines.

## Data Availability

The datasets presented in this article are not readily available because the sequencing data is from a cohort of patients and in order to protect their privacy, the data cannot be made publicly available. Requests to access the datasets should be directed to d.s.verbeek@umcg.nl.
