# Supplementary material for: Feasibility of Follow-Up Studies and Reclassification in Spinocerebellar Ataxia Gene Variants of Unknown Significance
Source: Front Genet. 2022 Mar 25;13:782685. doi: 10.3389/fgene.2022.782685 (PMC8990126; doi:10.3389/fgene.2022.782685)
Supplement: Supplementary file 1 [file DataSheet1.PDF]

Supplementary Table 1. List of SCA genes for targeted gene capturing panel.

SCA type	Gene name	Ref-seq	Type of mutation	Genome region	Reference
SCA1	*ATXN1*	NM_000332.3	CAG repeat expansion	Coding	(Orr et al., 1993)
SCA2	*ATXN2*	NM_002973.3	CAG repeat expansion	Coding	(Pulst et al., 1996; Sanpei et al., 1996)
SCA3	*ATXN3*	NM_004993.5	CAG repeat expansion	Coding	(Kawaguchi et al., 1994)
SCA5	*SPTBN2*	NM_006946.3	Missense, in-frame deletion	Coding	(Ikeda et al., 2006)
SCA6	*CACNA1A*	NM_023035.2	CAG repeat expansion	Coding	(Zhuchenko et al., 1997)
SCA7	*ATXN7*	NM_000333.3	CAG repeat expansion	Coding	(David et al., 1997)
SCA8	*ATXN8*	NR_002717.2	CTG repeat expansion	Non-coding	(Koob et al., 1999)
SCA10	*ATXN10*	NM_013236.3	ATTCT repeat expansion	Non-coding	(Matsuura et al., 2000)
SCA11	*TTBK2*	NM_173500.3	Frameshift	Coding	(Houlden et al., 2007)
SCA12	*PPP2R2B*	NM_001271899.1	CAG repeat	Non-coding	(Holmes et al., 1999)
SCA13	*KCNC3*	NM_004977.2	Missense	Coding	(Waters et al., 2006)
SCA14	*PRKCG*	NM_001316329.1	Missense	Coding	(Chen et al., 2003)
SCA15/16/29	*ITPR1*	NM_001168272.1	Missense, deletion	Coding	(Leemput et al., 2007; Iwaki et al., 2008; Huang et al., 2012)
SCA17	*TBP*	NM_003194.4	CAG repeat expansion	Coding	(Nakamura et al., 2001)
SCA19/22	*KCND3*	NM_004980.4	Missense	Coding	(Duarri et al., 2012; Lee et al., 2012)
SCA21	*TMEM240*	NM_001114748	Missense	Coding	(Delplanque et al., 2014)
SCA23	*PDYN*	NM_001190892.1	Missense	Coding	(Bakalkin et al., 2010)
SCA26	*eEF2*	NM_001961.3	Frameshift	Coding	(Hekman et al., 2012)
SCA27	*FGF14*	NM_001321939.1	Missense, frameshift	Coding	(van Swieten et al., 2003)
SCA28	*AFG3L2*	NM_006796.2	Missense	Coding	(Di Bella et al., 2010)
SCA31	*BEAN-TK2*	NM_001178020.2, NM_001271934.1	TGGAA repeat	Non-coding	(Sato et al., 2009)
SCA34	*ELOVL4*	NM_022726	Missense	Coding	(Cadieux-Dion et al., 2014)
SCA35	*TGM6*	NM_198994.2	Missense	Coding	(Wang et al., 2010)
SCA36	*NOP56*	NM_006392.3	GGCCTG repeat expansion	Non-coding	(Kobayashi et al., 2011)
SCA37	*DAB1*	NM_021080.4	ATTTC repeat expansion	Non-coding	(Seixas et al., 2017)
SCA38	*ELOVL5*	NM_001301856.1	Missense	Coding	(Di Gregorioet al., 2014)
SCA40	*CCDC88C*	NM_001080414.3	Missense	Coding	(Tsoi et al., 2014)
SCA41	*TRPC3*	NM_001130698.1	Missense	Coding	(Fogel et al., 2015)
SCA42	*CACNA1G*	NM_018896.4	Missense	Coding	(Coutelier et al., 2015)
SCA43	*MME*	NM_000902.3	Missense	Coding	(Depondt et al., 2016)
SCA44	*GRM1*	NM_001278064.1	Missense	Coding	(Watson et al., 2017)
SCA45	*FAT2*	NM_001447.2	Missense	Coding	(Nibbeling et al., 2017)
SCA46	*PLD3*	NM_012268.3	Missense	Coding	(Nibbeling et al., 2017)

-	*KIF26B*	NM_018012.4	Missense	Coding	(Nibbeling et al., 2017)
-	*EP300*	NM_001429.3	Missense	Coding	(Nibbeling et al., 2017)
-	*FAT1*	NM_005245.3	Missense	Coding	(Nibbeling et al., 2017)

Supplementary Table 2. The six *in silico* prediction programs – SIFT, PolyPhen2, MutationTaster, PhyloP, GranthamDistance and AlignGVGD – present in Alamut used to calculate the pathogenicity score. Per variant, each predictor can have a maximum score of 1 (damaging) and a minimum score of 0 (benign).

Prediction program	Benign (0)	Probably damaging (0.1)	Damaging (1)
SIFT	0	-	1
PolyPhen2	0	0.1	1
MutationTaster	0	-	1
PhyloP	0-2.49	2.5-3.0	>3
GranthamDistance	0-99	>100	>150
AlignGVGD	C0-C25	C35-C55	C65

Supplementary Table 3. Primers used to amplify the cDNAs generated by the wild type and mutant sequences of VUS-Splice and to introduce a VUS-Semi high and a VUS-High into plasmids with site-directed mutagenesis.

Gene	Variant	Classification	Forward primer	Reverse primer
CACNA1A	c.5157T>A	VUS-Splice	5'-CAGTGAATTCGGCACGTGGCATA-3'	5'-CAGTCTCGAGGCACGTCTTGCAT-3'
CACNA1G	c.3792G>T	VUS-Splice	5'-CAGTGAATTCGTGGGAGAGGGTAGACAA -3'	5'-CAGTGAGCTCGATCTGGTGAGAGACCTAAA-3'
FAT1	c.8991G>A	VUS-Splice	5'-CAGTGAATTCGCTGCCCTTGTTTT-3'	5'-CAGTCTCGAGTCGGCACTTTGTTT-3'
NOP56	c.909G>A	VUS-Splice	5'-CAGTGAATTCCTGTGCCTGGTCTG-3'	5'-CAGTCTCGAGTGCCAACCCATAGC-3'
TGM6	c.1171G>A: p.V391M	VUS-Semi high	5'-GATGGCCCCTTCATGTTTGCGGAGG-3'	5'-CCTCCGCAAACATGAAGGGGCCATC-3'
PRKCG	c.715C>T: p.R239W	VUS-High	5'-GATGTGGAGCGCTGGCTCAGCGTGG-3'	5'-CCACGCTGAGCCAGCGCTCCACATC-3'

Supplementary Table 4. Variants *in silico* predicted to have an effect on splicing by Alamut.

**Variant**	**Gene**	**Ref-seq**
c.909G>A	*NOP56*	NM_006392.3
c.608+3A>G	*ATXN3*	NM_004993.5
c.6400-11A>C	*CACNA1G*	NM_018896.4
c.484-7C>G	*CCDC88C*	NM_001080414.3
c.8991G>A	*FAT1*	NM_005245.3
c.7298A>G	*FAT1*	NM_005245.3
c.7463C>T	*FAT2*	NM_001447.2
c.1372-6T>C	*KCND3*	NM_004980.4
c.5157T>A	*CACNA1A*	NM_023035.2
c.897+22C>T	*EEF2*	NM_001961.3
c.214C>A	*EP300*	NM_001429.3
c.1417G>C	*ATXN7*	NM_000333.3
c.678-3_678-2insA	*TBP*	NM_003194.4
c.-22-10C>A	*SPTBN2*	NM_006946.3
c.3792G>T	*CACNA1G*	NM_018896.4
c.-19-1G>C	*PDYN*	NM_001190892.1
c.476-3_476-2insT	*ATXN3*	NM_004993.5

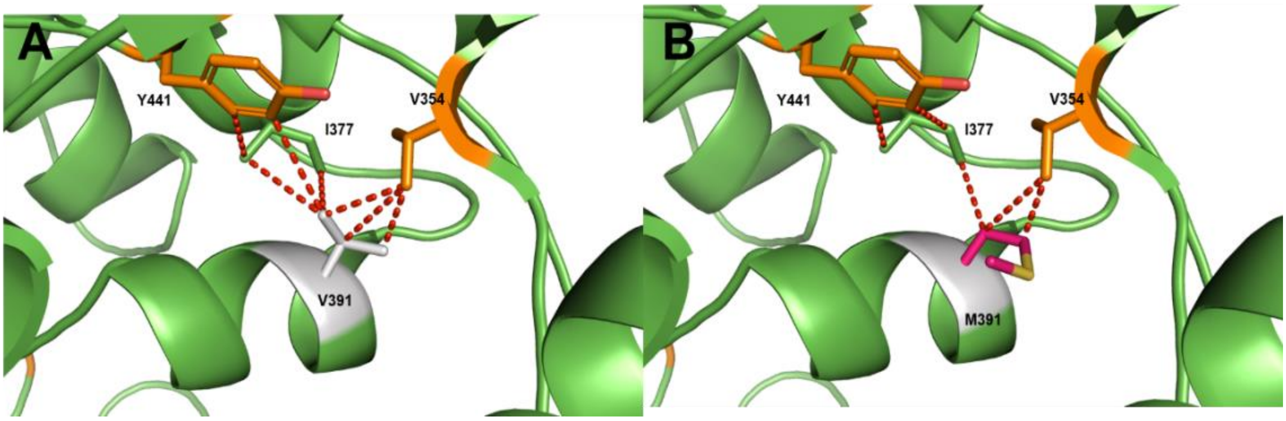

Supplementary Figure 1. V391M VUS-Semi high in TGM6 protein. (A) The native valine is shown as sticks with carbon atoms colored white. (B) Mutated methionine carbon atoms are colored pink. Known pathogenic mutations are colored orange. Hydrophobic bonds are shown as red dashes.

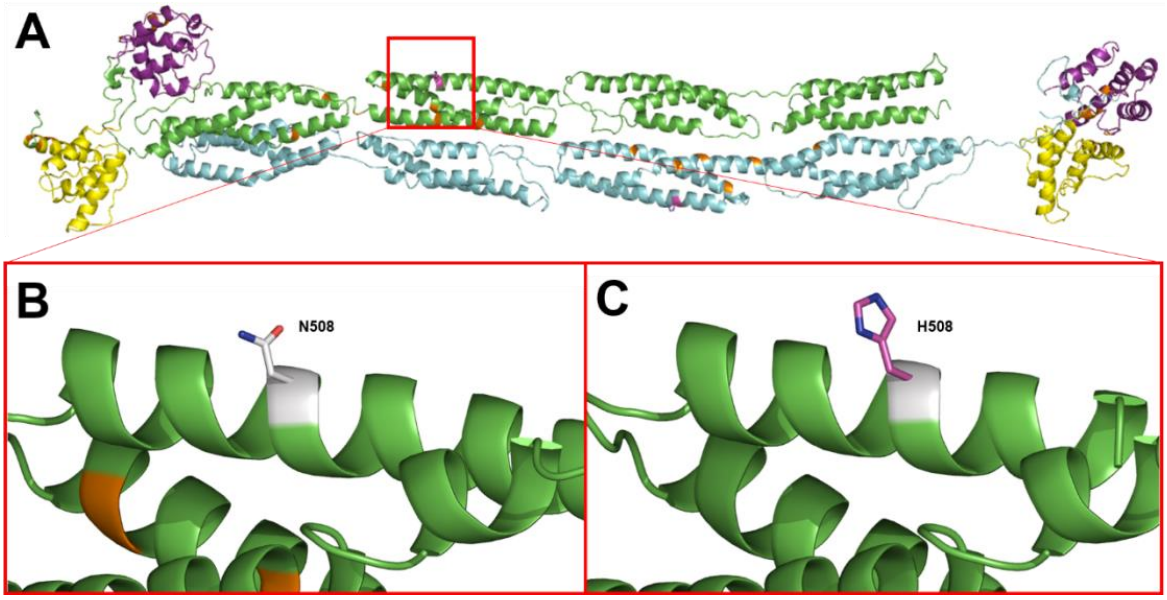

Supplementary Figure 2. Representation of N508H VUS-Semi high in beta-III spectrin protein. (A) The functional beta-III-spectrin is in a tetrameric complex composed of two alpha chains (green) and two beta chains (blue). The purple and yellow colors represent the Calponin Homology domain 1 (CH1) and Calponin Homology domain 2 (CH2) of the spectrin protein, respectively. (B) The native Asparagine side chain is shown as sticks with carbon atoms colored white. (C) Mutated histidine carbon atoms are colored pink. Known pathogenic mutations are colored orange. The histidine introduced by mutation N508H points towards the solvent area, similar to the native asparagine, indicating that the N508H variant has a minor impact on the protein structure.

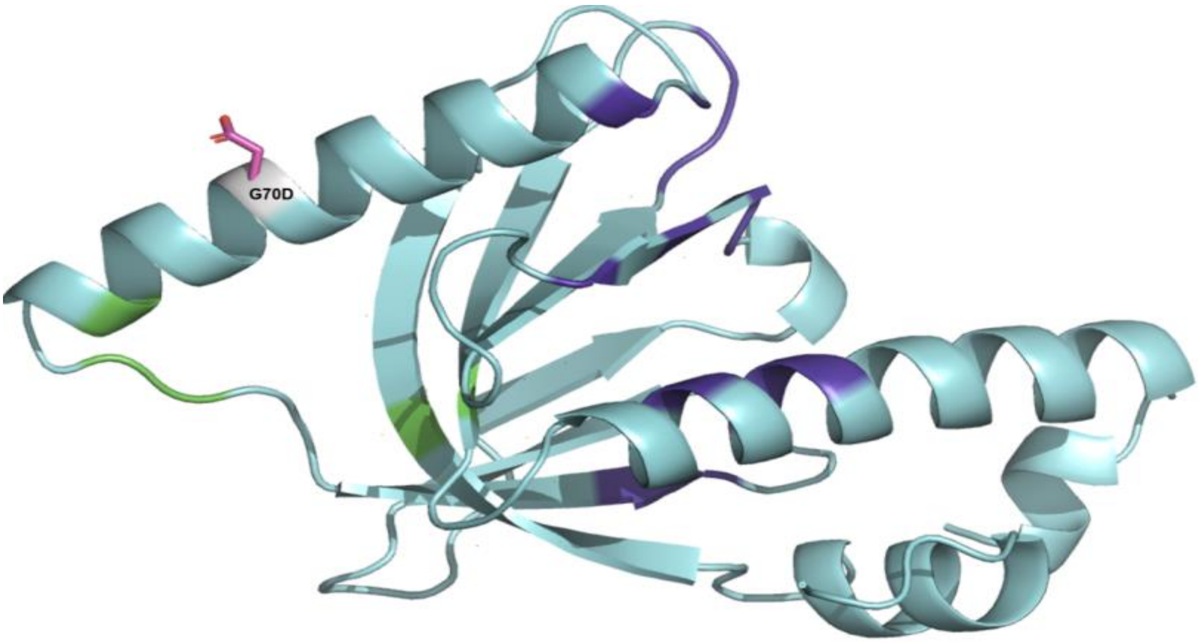

Supplementary Figure 3. G70D VUS-Semi high in DAB1 protein. The native glycine side chain is a hydrogen atom not shown in the figure. Mutated aspartic acid carbon atoms are colored pink. Residues forming the binding pocket for ApoER2 and phosphatidylinositol-4,5-biphosphate (PI-4,5-P_2) are shown as regions colored green and purple, respectively. The introduction of aspartic acid with G70D will lead to a positive charge but is located towards the solvent area. We hypothesize that it has only a minor effect on the protein structure.

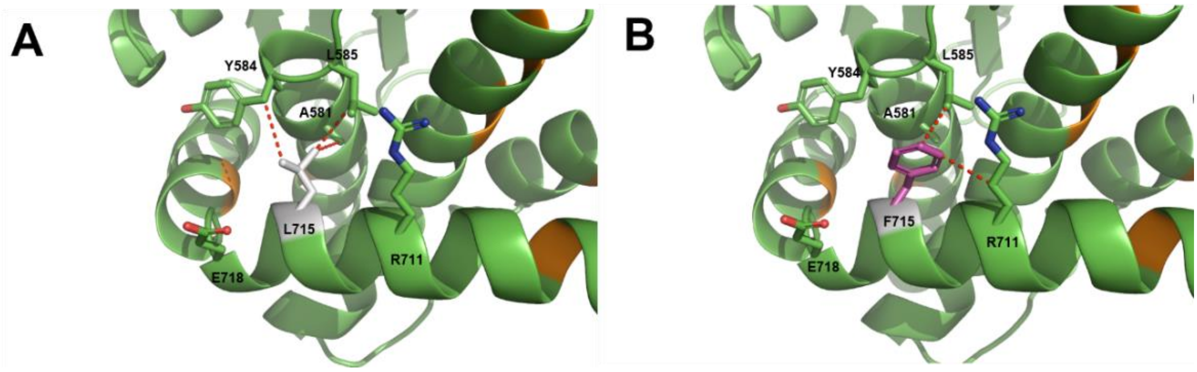

Supplementary Figure 4. L715F VUS-high in AFG3L2 protein. (A)The native leucine on position 715 is in contact with the hydrophobic residues A581, Y584 and L585. (B)The aromatic side chain of phenylalanine induced by L715F is stabilized by hydrophobic interactions with the side chain of L585 and R711. The native leucine is represented in white and the mutated phenylalanine in pink. Known pathogenic mutations are shaded orange. Hydrophobic bonds are shown as red dashes.

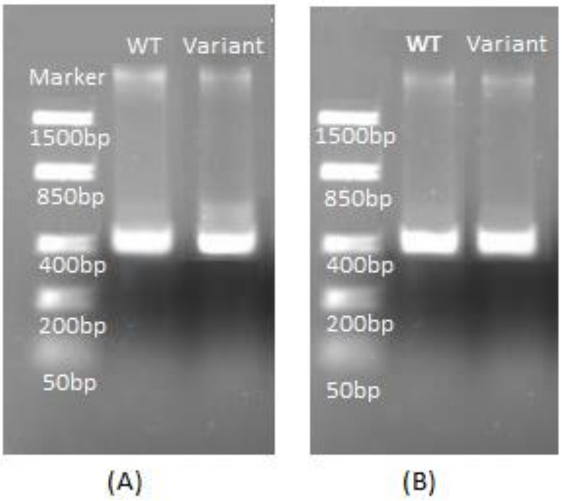

Supplementary Figure 5. DNA gel electrophoresis of PCR-amplified cDNA fragments generated from wild type and mutant sequences. (A) For *CACNA1A* (c.5157T>A) VUS-Splice, both the wild type and mutant fragments have the same size which is 445bp. (B) For *FAT1* (c.8991G>A) VUS-Splice, the wild-type and mutant fragment also do not show any difference in size and both are 460bp.

Supplementary results section

***TGM6* V391M VUS-Semi high**: The native valine residue at position 391 is located in a hydrophobic cluster with residues V354, Y441 and I377. Mutations on residues 354 and 441 are described as pathogenic variants with the insertion of isoleucine or cysteine, respectively. As shown in Supplementary figure 1, the side chain of the native V391 engages in a network of hydrophobic interactions with neighboring V354, Y441 and I377. The mutation V391M inserts a methionine at the position that contributes to the hydrophobic interaction in the pocket by its methylene carbons, thus conserving the native conformation. Due to the side chain contribution, the overall structure can be maintained and is thus functional.

***SPTBN2* N508H VUS-Semi high:** The N508H VUS-Semi high is located in the beta-III spectrin protein encoded by *SPTBN2* (isoform 1). The beta-III spectrin protein comprises 10 SPEC domains (Cho and Fogel, 2013). N508 is located in the second SPEC domain and is exposed to the solvent. In this domain, pathogenic variants such as R432S, M436T, R437Q/G, T472M, R480W and L491P have been reported. These pathogenic variants are in the intersection of the triple α-helical structure of the spectrin repeats and thus disrupt the structure of these repeats. Similar to the native asparagine, the histidine residue introduced by mutation N508H points towards the solvent area, in contrast to the other pathogenic variants mentioned, indicating that the N508H variant only has a minor impact on the protein structure (Supplementary figure 2).

***DAB1* G70D VUS-Semi high:** The native glycine at position 70 of DAB1 is located in the α2-helix, pointing toward the solvent area of the cytoplasm (isoform 1). The introduction of aspartic acid in this position will lead to a positive charge, but located towards the solvent area. Solvation layers are able to stabilize the mutated side chain, leading to its stabilization. In addition, the G70D is not in the binding region of the two known partners of DAB1, ApoER2 and phosphatidylinositol-4,5-biphosphate (PI-4,5-P), so the variant would not affect the binding of these partners (Supplementary figure 3). This variant is thus unlikely to influence protein function.

***AFG3L2* L715F VUS-High:** Three distinct models were built for the L715F variation in AFG3L2 (isoform 1). The three models are based on three available conformations of the formation unit of the AFG3L2 hexadimer. Chains B/C/D presented with an ATP analog (Phosphoaminophosphonic acid-adenylate ester, ANP) chain E, presenting an ADP molecule, and chains A/F in the apo form. The native leucine on position 715 is buried in a hydrophobic region in contact with residues A581, Y584 and L585 and is flanked by residues E718 and R711. The introduction of the aromatic side chain of phenylalanine is stabilized by hydrophobic interaction with the side chain of L585 and the methylene groups from the R711 side chain in models of chain A, B, D, E and F. In these models, the new arrangement of the residue F715 leads to a small cleft located between the F715 and the Y584, while the cleft between the position 715 and R711 no longer exists. The only difference observed was for the chain C model, where the mutated phenylalanine points towards Y584 and conserves the cleft between itself and the R711. This leads to the hypothesis that position 715 can accommodate different residues, indicating tolerance to variants such as the L715F. In addition, the L517F variant does not cause major displacements in essential areas such as the substrate-binding area, zinc-binding region, catalytic region, or the intersection between chains, which further rules out a damaging effect of L715F. Finally, L715F is not located in any of the previously reported mutation hotspots of *AFG3L2* (Puchades et al., 2019) and is not in contact with any of the known pathogenic variants. We therefore do not deem this variant pathogenic (Supplementary figure 4).

References:

H.T. Orr, M.-Y. Chung, S. Banfi, T.J. Kwiatkowski, A. Servadio, A.L. Beaudet, et al., Expansion of an unstable trinucleotide CAG repeat in spinocerebellar ataxia type 1, Nat. Genet. 4 (1993) 221–226. doi:10.1038/ng0793-221.

S.M. Pulst, A. Nechiporuk, T. Nechiporuk, S. Gispert, X.-N. Chen, I. Lopes-Cendes, et al., Moderate expansion of a normally biallelic trinucleotide repeat in spinocerebellar ataxia type 2, Nat. Genet. 14 (1996) 269–276. doi:10.1038/ng1196-269.

K. Sanpei, H. Takano, S. Igarashi, T. Sato, M. Oyake, H. Sasaki, et al., Identification of the spinocerebellar ataxia type 2 gene using a direct identification of repeat expansion and cloning technique, DIRECT, Nat. Genet. 14 (1996) 277–284. doi:10.1038/ng1196-277.

Y. Kawaguchi, T. Okamoto, M. Taniwaki, M. Aizawa, M. Inoue, S. Katayama, et al., CAG expansions in a novel gene for Machado-Joseph disease at chromosome 14q32.1, Nat. Genet. 8 (1994) 221–228. doi:10.1038/ng1194-221.

Y. Ikeda, K.A. Dick, M.R. Weatherspoon, D. Gincel, K.R. Armbrust, J.C. Dalton, et al., Spectrin mutations cause spinocerebellar ataxia type 5, Nat. Genet. 38 (2006) 184–190. doi:10.1038/ng1728.

O. Zhuchenko, J. Bailey, P. Bonnen, T. Ashizawa, D.W. Stockton, C. Amos, et al., Autosomal dominant cerebellar ataxia (SCA6) associated with small polyglutamine expansions in the alpha 1A-voltage-dependent calcium channel, Nat. Genet. 15 (1997) 62–69. doi:10.1038/ng0197-62.

G. David, N. Abbas, G. Stevanin, A. Durr, G. Yvert, G. Cancel, et al., Cloning of the SCA7 gene reveals a highly unstable CAG repeat expansion, Nat. Genet. 17 (1997) 65–70. doi:10.1038/ng0997-65.

M.D. Koob, M.L. Moseley, L.J. Schut, K.A. Benzow, T.D. Bird, J.W. Day, et al., An untranslated CTG expansion causes a novel form of spinocerebellar ataxia (SCA8), Nat. Genet. 21 (1999) 379–384. doi:10.1038/7710.

T. Matsuura, T. Yamagata, D.L. Burgess, A. Rasmussen, R.P. Grewal, K. Watase, et al., Large expansion of the ATTCT pentanucleotide repeat in spinocerebellar ataxia type 10, Nat. Genet. 26 (2000) 191–194. doi:10.1038/79911.

H. Houlden, J. Johnson, C. Gardner-Thorpe, T. Lashley, D. Hernandez, P. Worth, et al., Mutations in TTBK2, encoding a kinase implicated in tau phosphorylation, segregate with spinocerebellar ataxia type 11, Nat. Genet. 39 (2007) 1434–1436. doi:10.1038/ng.2007.43.

S.E. Holmes, E.E. O'Hearn, M.G. McInnis, D.A. Gorelick-Feldman, J.J. Kleiderlein, C. Callahan, et al., Expansion of a novel CAG trinucleotide repeat in the 5' region of PPP2R2B is associated with SCA12, Nat. Genet. 23 (1999) 391–392. doi:10.1038/70493.

M.F. Waters, N.A. Minassian, G. Stevanin, K.P. Figueroa, J.P.A. Bannister, D. Nolte, et al., Mutations in voltage-gated potassium channel KCNC3 cause degenerative and developmental central nervous system phenotypes, Nat. Genet. 38 (2006) 447–451. doi:10.1038/ng1758.

D.-H. Chen, Z. Brkanac, C.L.M.J. Verlinde, X.-J. Tan, L. Bylenok, D. Nochlin, et al., Missense mutations in the regulatory domain of PKC gamma: a new mechanism for dominant nonepisodic cerebellar ataxia, Am. J. Hum. Genet. 72 (2003) 839– 849.

J. van de Leemput, J. Chandran, M.A. Knight, L.A. Holtzclaw, S. Scholz, M.R. Cookson, et al., Deletion at ITPR1 underlies ataxia in mice and spinocerebellar ataxia 15 in humans, PLoS Genet. 3 (2007) e108. doi:10.1371/journal.pgen.0030108.

A. Iwaki, Y. Kawano, S. Miura, H. Shibata, D. Matsuse, W. Li, et al., Heterozygous deletion of ITPR1, but not SUMF1, in spinocerebellar ataxia type 16, J. Med. Genet. 45 (2008) 32–35. doi:10.1136/jmg.2007.053942.

L. Huang, J.W. Chardon, M.T. Carter, K.L. Friend, T.E. Dudding, J. Schwartzentruber, et al., Missense mutations in ITPR1 cause autosomal dominant congenital nonprogressive spinocerebellar ataxia, Orphanet J Rare Dis. 7 (2012) 67. doi:10.1186/1750-1172-7-67. ACC.

K. Nakamura, S.Y. Jeong, T. Uchihara, M. Anno, K. Nagashima, T. Nagashima, et al., SCA17, a novel autosomal dominant cerebellar ataxia caused by an expanded polyglutamine in TATA-binding protein, Hum. Mol. Genet. 10 (2001) 1441–1448.

A. Duarri, J. Jezierska, M. Fokkens, M. Meijer, H.J. Schelhaas, W.F.A. den Dunnen, et al., Mutations in potassium channel kcnd3 cause spinocerebellar ataxia type 19, Ann. Neurol. 72 (2012) 870–880. doi:10.1002/ana.23700.

Y.-C. Lee, A. Durr, K. Majczenko, Y.-H. Huang, Y.-C. Liu, C.-C. Lien, et al., Mutations in KCND3 cause spinocerebellar ataxia type 22, Ann. Neurol. 72 (2012) 859–869. doi:10.1002/ana.23701.

J. Delplanque, D. Devos, V. Huin, A. Genet, O. Sand, C. Moreau, et al., TMEM240 mutations cause spinocerebellar ataxia 21 with mental retardation and severe cognitive impairment, Brain. 137 (2014) 2657–2663. doi:10.1093/brain/awu202.

G. Bakalkin, H. Watanabe, J. Jezierska, C. Depoorter, C. Verschuuren-Bemelmans, I. Bazov, et al., Prodynorphin mutations cause the neurodegenerative disorder spinocerebellar ataxia type 23, Am. J. Hum. Genet. 87 (2010) 593–603. doi:10.1016/j.ajhg.2010.10.001.

K.E. Hekman, G.-Y. Yu, C.D. Brown, H. Zhu, X. Du, K. Gervin, et al., A conserved eEF2 coding variant in SCA26 leads to loss of translational fidelity and increased susceptibility to proteostatic insult, Hum. Mol. Genet. 21 (2012) 5472–5483. doi:10.1093/hmg/dds392.

J.C. van Swieten, E. Brusse, B.M. de Graaf, E. Krieger, R. van de Graaf, I. de Koning, et al., A Mutation in the Fibroblast Growth Factor 14 Gene Is Associated with Autosomal Dominant Cerebral Ataxia, The American Journal of Human Genetics. 72 (2003) 191–199. doi:10.1086/345488.

D. Di Bella, F. Lazzaro, A. Brusco, M. Plumari, G. Battaglia, A. Pastore, et al., Mutations in the mitochondrial protease gene AFG3L2 cause dominant hereditary ataxia SCA28, Nat. Genet. 42 (2010) 313–321. doi:10.1038/ng.544.

N. Sato, T. Amino, K. Kobayashi, S. Asakawa, T. Ishiguro, T. Tsunemi, et al., Spinocerebellar ataxia type 31 is associated with “inserted” penta-nucleotide repeats containing (TGGAA)n, Am. J. Hum. Genet. 85 (2009) 544–557. doi:10.1016/j.ajhg.2009.09.019.

M. Cadieux-Dion, M. Turcotte-Gauthier, A. Noreau, C. Martin, C. Meloche, M. Gravel, et al., Expanding the clinical phenotype associated with ELOVL4 mutation: study of a large French-Canadian family with autosomal dominant spinocerebellar ataxia and erythrokeratodermia, JAMA Neurol. 71 (2014) 470–475. doi:10.1001/jamaneurol.2013.6337.

J.-L. Wang, X. Yang, K. Xia, Z.M. Hu, L. Weng, X. Jin, et al., TGM6 identified as a novel causative gene of spinocerebellar ataxias using exome sequencing, Brain. 133 (2010) 3510–3518. doi:10.1093/brain/awq323.

H. Kobayashi, K. Abe, T. Matsuura, Y. Ikeda, T. Hitomi, Y. Akechi, et al., Expansion of intronic GGCCTG hexanucleotide repeat in NOP56 causes SCA36, a type of spinocerebellar ataxia accompanied by motor neuron involvement, Am. J. Hum. Genet. 89 (2011) 121–130. doi:10.1016/j.ajhg.2011.05.015.

A.I. Seixas, J.R. Loureiro, C. Costa, A. Ordóñez-Ugalde, H. Marcelino, C.L. Oliveira, et al., A Pentanucleotide ATTTC Repeat Insertion in the Non-coding Region of DAB1, Mapping to SCA37, Causes Spinocerebellar Ataxia, Am. J. Hum. Genet. 101 (2017) 87–103. doi:10.1016/j.ajhg.2017.06.007.

E. Di Gregorio, B. Borroni, E. Giorgio, D. Lacerenza, M. Ferrero, N. Lo Buono, et al., ELOVL5 mutations cause spinocerebellar ataxia 38, Am. J. Hum. Genet. 95 (2014) 209–217. doi:10.1016/j.ajhg.2014.07.001.

H. Tsoi, A.C.S. Yu, Z.S. Chen, N.K.N. Ng, A.Y.Y. Chan, L.Y.P. Yuen, et al., A novel missense mutation in CCDC88C activates the JNK pathway and causes a dominant form of spinocerebellar ataxia, J. Med. Genet. 51 (2014) 590–595. doi:10.1136/jmedgenet-2014-102333.

B.L. Fogel, S.M. Hanson, E.B.E. Becker, Do mutations in the murine ataxia gene TRPC3 cause cerebellar ataxia in humans? Mov. Disord. 30 (2015) 284–286. doi:10.1002/mds.26096.

M. Coutelier, I. Blesneac, A. Monteil, M.-L. Monin, K. Ando, E. Mundwiller, et al., A Recurrent Mutation in CACNA1G Alters Cav3.1 T-Type Calcium-Channel Conduction and Causes Autosomal-Dominant Cerebellar Ataxia, Am. J. Hum. Genet. 97 (2015) 726–737. doi:10.1016/j.ajhg.2015.09.007.

C. Depondt, S. Donatello, M. Rai, F.C. Wang, M. Manto, N. Simonis, et al., MME mutation in dominant spinocerebellar ataxia with neuropathy (SCA43), Neurol Genet. 2 (2016) e94. doi:10.1212/NXG.0000000000000094.

L.M. Watson, E. Bamber, R.P. Schnekenberg, J. Williams, C. Bettencourt, S. Jayawant, et al., Dominant Mutations in GRM1 Cause Spinocerebellar Ataxia Type 44, Am. J. Hum. Genet. 101 (2017) 638. doi:10.1016/j.ajhg.2017.09.006.

E.A.R. Nibbeling, A. Duarri, C.C. Verschuuren-Bemelmans, M.R. Fokkens, J.M. Karjalainen, C.J.L.M. Smeets, et al., Exome sequencing and network analysis identifies shared mechanisms underlying spinocerebellar ataxia, Brain. (2017). doi:10.1093/brain/awx251.

Cho E, Fogel BL. A family with spinocerebellar ataxia type 5 found to have a novel missense mutation within a SPTBN2 spectrin repeat. The Cerebellum. 2013 Apr 1;12(2):162-4.

Puchades C, Ding B, Song A, Wiseman RL, Lander GC, Glynn SE. Unique structural features of the mitochondrial AAA+ protease AFG3L2 reveal the molecular basis for activity in health and disease. Molecular cell. 2019 Sep 5;75(5):1073-85.
